# Effects of Time-Interval since Blood Draw and of Anticoagulation on Platelet Testing (Count, Indices and Impedance Aggregometry): A Systematic Study with Blood from Healthy Volunteers

**DOI:** 10.3390/jcm9082515

**Published:** 2020-08-04

**Authors:** Michael Hardy, Sarah Lessire, Sultan Kasikci, Justine Baudar, Maité Guldenpfennig, Adrien Collard, Jean-Michel Dogné, Bernard Chatelain, Hugues Jacqmin, Thomas Lecompte, François Mullier

**Affiliations:** 1 CHU UCL Namur, Namur Thrombosis and Hemostasis Center (NTHC), Namur Research Institute for Life Sciences (NARILIS), Hematology Laboratory, Université catholique de Louvain (UCL), 5530 Yvoir, Belgium; sultan.kasikci@uclouvain.be (S.K.); justine.baudar@uclouvain.be (J.B.); maite.guldenpfennig@uclouvain.be (M.G.); adrien.collard@uclouvain.be (A.C.); bernard.chatelain@uclouvain.be (B.C.); hugues.jacqmin@uclouvain.be (H.J.); francois.mullier@uclouvain.be (F.M.); 2 CHU UCL Namur, Namur Thrombosis and Hemostasis Center (NTHC), Namur Research Institute for Life Sciences (NARILIS), Department of Anesthesiology, Université catholique de Louvain (UCL), 5530 Yvoir, Belgium; sarah.lessire@uclouvain.be; 3 Department of Pharmacy, Namur Thrombosis and Hemostasis Center (NTHC), Namur Research Institute for Life Sciences (NARILIS), University of Namur, 5000 Namur, Belgium; jean-michel.dogne@unamur.be; 4 Department of Medicine, Angiology and Hemostasis Division, Geneva University Hospitals, and Geneva Platelet Group (GpG), Faculty of Medicine, University of Geneva, 14 CH-1211 Geneva, Switzerland; thomaspierre.lecompte@hcuge.ch

**Keywords:** platelet count, MPV, IPF, multiplate, platelet function, aggregometry, preanalytical, anticoagulant, citrate, hirudin

## Abstract

Platelet count, indices (mean volume, young—immature platelet fraction) and aggregation are widely used laboratory parameters to investigate primary hemostasis. We performed a systematic, thorough evaluation of the influence of the time-interval since blood draw from 20 healthy individuals and of the anticoagulation of collected blood on such parameters. Blood was anticoagulated with citrate, K_2_-ethylenediaminetetraacetic acid (EDTA) and hirudin and analyzed 5, 30, 60, 120 and 180 min after blood draw. Multiple electrode aggregometry (MEA) was performed with either hirudin (half-diluted with NaCl) or citrate samples (half-diluted with NaCl or CaCl_2_ 3 mM). Platelet count and indices (Sysmex XN-20) were rather stable over time with EDTA blood. MEA results were lower with citrate blood than with hirudin blood; supplementation with calcium was partially compensatory. MEA results were also lower when performed less than 30 or more than 120 min after blood draw. Platelet clumping, quantitatively estimated with microscope examination of blood smears, was more important in hirudin blood than citrate or EDTA blood and could explain some of the differences observed between preanalytical variables. The results stress once more the importance of preanalytical variables in hemostasis laboratory testing. Decision thresholds based on those tests are only applicable within specific preanalytical conditions.

## 1. Introduction

Platelets are a central component of hemostasis under normal and pathological conditions and play an important role in both hemorrhagic and thrombotic disorders. Therefore, platelet tests can be critical for clinical decision-making. Indeed, platelet count is an essential tool for the diagnosis and follow-up of thrombocytopenic diseases and a main trigger of platelet transfusion in hemorrhagic disorders. Increased mean platelet volume (MPV) has been used for differential diagnosis of thrombocytopenia and as a biomarker of prothrombotic states, cardiovascular diseases and mortality in patients with sepsis, to name a few potential clinical applications [1,2,3]. The immature platelet fraction (IPF) is deemed a surrogate of bone marrow production of platelets. It has been used to differentiate peripheral and central causes of thrombocytopenia and to identify the resumption of thrombopoiesis after myelosuppression, for example to decide whether platelet transfusion was necessary [4,5,6]. Platelet function testing (PFT) has been used to individually tailor antiplatelet drug therapy in high-risk patients, to ensure the absence of residual effect of antiplatelet drugs before surgery or to screen for platelet function disorders [7,8,9]. As all those laboratory parameters have been used for clinical diagnosis, prognosis and treatment, their accurate measurement is essential for reliable clinical management.

The preanalytical phase is the most critical part of the analyzing process in laboratory hemostasis [10]. Several parameters such as blood collection technique, processing, transport, storage conditions and duration can influence the results and contribute to increase both intra- and inter-laboratory variability [10]. One specific issue regarding platelets is their propensity to stick together in the collected blood sample, whatever the mechanism is—genuine aggregation or mere agglutination [11]; in the following text, we will use the word “clump” to remain purely descriptive. Clinical guidelines usually lack details on the suitable preanalytical conditions [7,12]. For instance, for PFT using multiple electrode impedance aggregometry (MEA), the manufacturer recommends performing the analysis between 30 and 180 min after blood draw and to use hirudin containing tubes, as chelation of calcium ions by citrate would reduce platelet aggregation [13]. However, in clinical practice, citrate tubes are often used. Some proposed to supplement the sample with CaCl_2_ to mitigate the effect of calcium chelation on platelet reactivity, even if few data support this practice [14]. For complete blood count and derived indices, K_2_- or K_3_-ethylenediaminetetraacetic acid (EDTA) tubes are recommended [15]. However, no specific time-interval between blood draw and measurement has been clearly specified. The influence of anticoagulants or time-intervals since blood draw on platelet count, indices and function measurement has only been sparsely studied so far, with sometimes conflicting results. The impact of these variables should therefore be studied more comprehensively in order to define the optimal preanalytical conditions. This could minimize the variability of the measurements and increase the relevance of resulting clinical decisions.

The aim of this study was to assess the effect of two preanalytical variables, the type of anticoagulant used and the time elapsed between blood draw and analysis on platelet count (impedance (PLT-I)), so-called optical (PLT-O) method, fluorescence (PLT-F) method [16]) and MPV (impedance method), IPF, all measured with a Sysmex XN-20 cell counter, and MEA testing. We also explored whether the platelet count or the formation of platelet clumps and its extent could explain at least part of the differences observed for those tests between preanalytical conditions.

## 2. Materials and Methods

This study was approved by the Ethics Committee of the CHU UCL Namur (NUB: B03920096633). Twenty healthy volunteers have been included after providing written informed consent. Individuals who had taken aspirin, nonsteroidal anti-inflammatory drugs or any other drug that could alter platelet function in the previous 10 days were excluded. All blood samples were drawn and analyzed at the CHU UCL Namur (Godinne site, Yvoir, Belgium).

Blood was drawn directly within the central laboratory by venipuncture of an antebrachial vein using 20 G needles (Terumo^®^, Tokyo, Japan). The following order was used: one discard tube for the first milliliters of blood, four 3.5 mL plastic tubes (polypropylene) containing sodium citrate 109 mM (1:9 v/v) as anticoagulant (Vacuette^®^, Greiner Bio One^®^, Kremsmünster, Austria), one 4 mL plastic tube (polyethylene terephtalate) containing dried sprayed K_2_-ethylenediaminetetraacetic acid (EDTA) as anticoagulant (Vacuette^®^, Greiner Bio One^®^, Kremsmünster, Austria) and five 3 mL plastic tubes (polypropylene) containing dried sprayed recombinant hirudin as anticoagulant (concentration 25 μg/mL; Roche diagnostics, Basel, Switzerland). The tourniquet was kept in place for less than 60 s and released during the filling of the discard tube. Three gentle inversions of the tubes were performed to ensure the proper mixing of blood with the anticoagulant. Platelet count, indices (MPV and IPF) and aggregation were analyzed at five time points after blood draw—5, 30, 60, 120 and 180 min. Blood samples were stored statically at room temperature (22.8 °C ± 0.5 during the study period) between the tests and homogenized with eight gentle inversions before each test.

**Figure 1:**
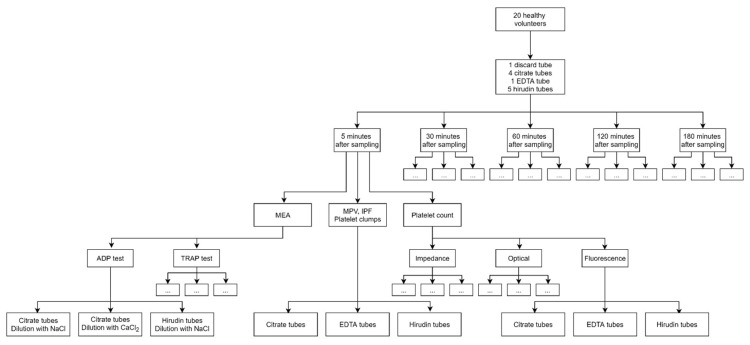
Study design. Empty boxes just repeat the same elements as adjacent boxes. MEA: multiple electrode aggregometry; MPV: mean platelet volume; IPF: immature platelet fraction; EDTA: ethylenediaminetetraacetic acid; ADP: adenosine diphosphate; TRAP: thrombin receptor activating peptide.

Platelet count and platelet indices (MPV, IPF) were measured at the same five time points with blood anticoagulated either with sodium citrate, K_2_-EDTA or hirudin (Figure 1). Measurements were performed with a Sysmex^®^ XN-20 analyzer (Sysmex^®^, Kobe, Japan; IPU version 22.00-00) according to manufacturer’s recommendations. Platelet count was determined with impedance, so-called optical (RET—reticulocyte—channel, using polymethine as dye) and fluorescence (specific channel and reagents dedicated to platelet counting, using oxazine as dye) methods and expressed as G/L [17,18]. Platelet counts of citrate blood were corrected by multiplying the result by 1/0.9 to account for the dilution of the sample. MPV (measured in fL) was determined with impedance method and IPF (expressed as the ratio of young platelets to total number of platelets) with the fluorescence method. The Sysmex flags and Qflags (relative scale from 0 to 300) for the presence of platelet clumps were also recorded.

Platelet aggregation was studied in whole blood with MEA using two Multiplate^®^ analyzers according to manufacturer’s recommendations (Roche diagnostics, Basel, Switzerland) [19,20]. Briefly, 300 μL of whole blood were incubated with 300 μL of dilution solution (see below) to optimize the impedance measurement [21]. Then, platelets were activated with adenosine diphosphate (ADP; final concentration: 6.5 μM; ADP test) or thrombin receptor activating peptide 6 (TRAP; final concentration: 32 μM; TRAP test). All reagents were provided by the manufacturer (Roche diagnostics). Platelet response was evaluated by the area under the curve of the impedance changes over time and expressed in arbitrary units (U). Three anticoagulation conditions were simultaneously tested (tests were run on three wells in parallel): (i) hirudin in the sample tube, diluted with NaCl 154 mM 1:1 v/v before analysis, (ii) sodium citrate in the sample tube, diluted with NaCl 154 mM 1:1 v/v before analysis, and (iii) sodium citrate in the sample tube, diluted with NaCl 154 mM + CaCl_2_ 3 mM 1:1 v/v before analysis. At each time point, 2 MEA tests (with ADP and TRAP) were performed for each blood sample and each anticoagulation condition, resulting in 30 analyses per subject and a total of 600 analyses for the 20 subjects (Figure 1).

Finally, a quantitative estimate of platelet clumping was made by counting platelets remained isolated and those in clumps with optical microscopy (OM) at each time point, for the three types of anticoagulated blood used for platelet count and indices (citrate, EDTA or hirudin), by the same operator. Blood was smeared onto a glass slide and dried, and eventually colored using May–Grunwald–Giemsa stain. Isolated and clumped platelets were counted at the feather edge in ten adjacent fields using a Miller reticle [11]. We calculated the relative proportion of clumped platelets to the total number of platelets (sum of clumped and isolated ones). This index was chosen because it is independent of the slide preparation technique (spreading of blood components).

All analyses were performed with the same batches of reagents. The same tube was used for the measurement of platelet count, platelet indices (MPV, IPF) and platelet clumps. Four hirudin tubes and three citrate tubes were used per patient to perform MEA analysis. Blood from those tubes has not been pooled to avoid an additional preanalytical step that could have impacted the result.

### Statistical Analysis

Mean differences (and relative differences (%)) between pre-analytical conditions are given with their 95% confidence intervals. The effect of the two study variables was evaluated using a linear mixed model accounting for the random effect of subjects [22]. A subject-specific intercept was used for modeling the effect of the anticoagulant and the time-interval between blood draw and analysis on each MEA test (ADP and TRAP). The fit of the model was evaluated graphically. The global effect of both study variables, as well as differences between time points and anticoagulation conditions were estimated by likelihood ratio (LR) tests. The same methodology was applied to platelet count (with the method of platelet counting—impedance, optical or fluorescence—as a third variable), to MPV and IPF measurements. Then, we evaluated whether the platelet count could explain baseline differences of MPV, IPF and platelet reactivity between subjects (using conventional linear models), and whether the percentage of platelet within clumps could explain variations of platelet count, MPV and IPF observed between time-intervals and anticoagulants (using linear mixed models as previously).

To evaluate the clinical impact of the differences observed between preanalytical conditions, we calculated the reference change values (RCV) for each test (CVa: analytical coefficient of variation, CVi: intra-individual coefficient of variation) [23]:

RCV = 1.96 × √2 × √(CVa^2^ + CVi^2^) (1)

As compared analyses were performed with the same blood draw under different pre-analytical conditions, CVi is nonexistent and RCV formula can be simplified by eliminating intra-individual variability (CVi = 0). The clinically acceptable difference between two measurements of the same sample is then called the critical difference (CD) [24]:

CD = 1.96 × √(2 × CVa^2^) = 2.77 × CVa (2)

For platelet count, MPV and IPF, CVas were calculated from daily quality controls performed on the Sysmex analyzer (*n* = 62). For MEA, we calculated CVa from quintuplicate tests performed on eight different samples using the same batches of reagents as those used in the present study. Based on these CVas, CDs were 6% for platelet count, 4% for MPV, 9% for IPF, 19% for ADP-MEA and 12% for TRAP-MEA. The changes were thus defined as clinically acceptable if the observed mean differences were lower than predetermined CDs.

The sample size required was been calculated for MEA analysis. Based on internal validation data, we calculated that a sample size of 20 patients would be enough to identify differences of at least 10% (for TRAP MEA) or 15% (for ADP MEA) between the different preanalytical conditions we studied, with a power greater than 80% (for alpha = 0.05). All statistical analyses were performed in R (version 3.6.1) [25], using nlme package (version 3.1–140); ggplot2 package (version 3.2.1) was used to construct graphs.

## 3. Results

Twenty healthy volunteers were enrolled in this study (median age 35 years (from 21 to 61), 16/20 females). For one subject, PLT-I and MPV measurements and MEA analysis were not feasible at 180 min due to too low residual blood volume in hirudin tubes.

### 3.1. Platelet Count

**Figure 2:**
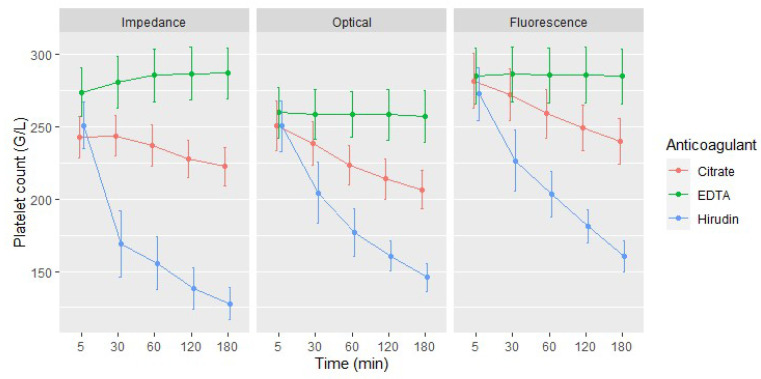
Mean platelet count (G/L) according to the time elapsed between blood draw and analysis. Red lines represent tests performed with blood samples anticoagulated with citrate, green lines with EDTA and blue lines with hirudin. Bars represent 95% CI of the mean.

Globally, we observed a statistically and clinically significant reduction in apparent platelet count as time from blood draw passed (*p* < 0.001, LR test). This effect was more pronounced for hirudin blood than for citrate blood and was negligible with EDTA blood (Figure 2). For example, platelet count at 60 min was lowered by 19 G/L (95% CI: 8–29) and 79 G/L (95% CI: 69–89), compared to platelet count at 5 min in citrate and hirudin blood samples, respectively (Table S1).

We also identified a statistically and clinically significant difference of apparent platelet counts between the three anticoagulants (*p* < 0.001, LR test), except when analysis was performed within 5 min after blood draw by optical or fluorescence methods (Table S2). Platelet count was indeed higher when measured with EDTA blood, by comparison with citrate blood, and lower with hirudin blood, by comparison with the latter. The effect of the anticoagulants was also significantly different for the different methods of measurement (*p* < 0.001, LR test) (Figure 2). Of note, the fluorescence method measured higher platelet counts than the two others (Table S3).

**Figure 3:**
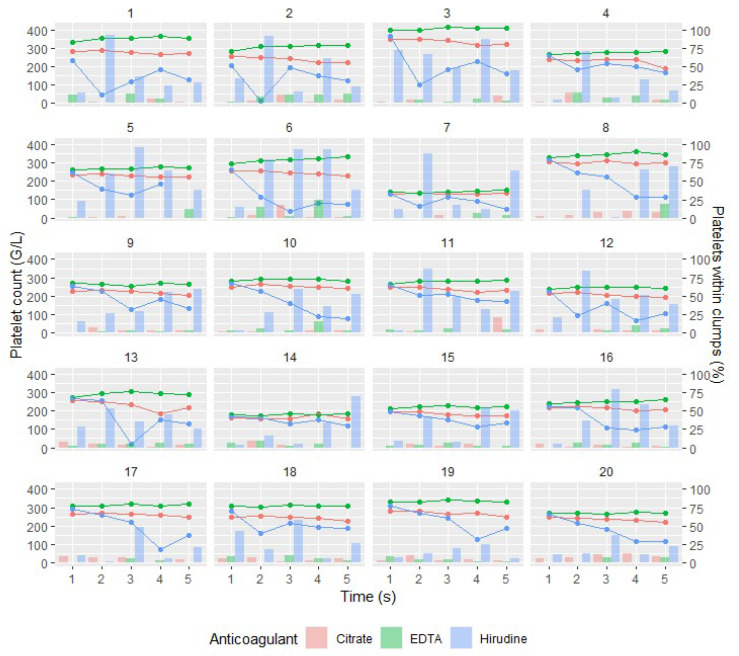
Platelet count measured by impedance method (lines) and percentage of platelets within clumps (bars) over time for each subject (*n* = 20) and anticoagulant. Red lines/bars represent tests performed with blood samples anticoagulated with citrate, green lines/bars with EDTA and blue lines/bars with hirudin. We can observe that platelet count is partly varying in relation to the percentage of platelets within clumps.

We found a significant association between platelet count and percentage of platelets within clumps (*p* < 0.001, LR test; Figure 3). The effect of platelet clumps on platelet count was more pronounced when platelets were counted by impedance method than when they were counted by optical or fluorescence methods (*p* < 0.001, LR test).

### 3.2. Platelet Indices

**Figure 4:**
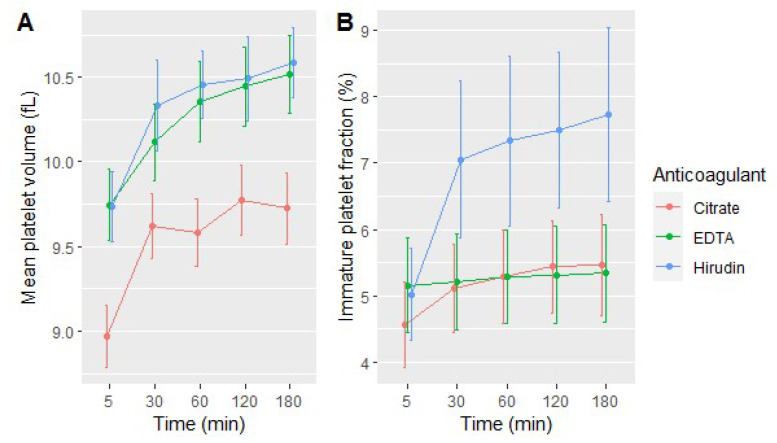
Mean platelet volume (**A**) or mean immature platelet fraction (**B**) according to the time elapsed between blood draw and analysis. Red lines represent tests performed with blood samples anticoagulated with citrate, green lines with EDTA and blue lines with hirudin. Bars represent 95% CI of the mean.

Globally, the type of anticoagulant had a statistically and clinically significant effect on MPV and IPF measurements (*p* < 0.001, LR test; Figure 4). MPV measured with citrated blood was lower than MPV measured with EDTA or hirudin blood (*p* < 0.001, LR test). For example, MPV measured 60 min after blood draw with citrate blood was 0.77 and 0.87 fL lower than MPV measured with EDTA and hirudin blood, respectively (Table S4). However, there was no statistically significant difference between EDTA and hirudin samples. With blood drawn into hirudin tubes, there was a higher IPF than blood drawn into citrate and K_2_-EDTA tubes (*p* < 0.001, LR test; Figure 4B). For example, IPF measured 60 min after blood draw with hirudin blood was 2% higher than IPF measured with EDTA and citrate blood (Table S5). There was no statistically significant difference in IPF measurements between citrate and EDTA blood.

We also observed a statistically and clinically significant increase in MPV and IPF measurement over time (*p* < 0.001, LR test), except for IPF when blood was anticoagulated with EDTA (Table 1 and Table 2).

At baseline, we identified a significant increase in MPV and IPF with decreasing platelet counts (*p* < 0.001, Student *t*-test). On the whole, there was also a significant association between the percentage of platelets within clumps and both MPV and IPF (*p* < 0.001, LR test).

### 3.3. Platelet Function—MEA

Both pre-analytical conditions studied (i.e., anticoagulation and time elapsed between blood draw and analysis) significantly influenced MEA result (*p* < 0.001, LR test). The differences among anticoagulation conditions were not the same at the different time points studied (*p* < 0.001, LR test).

**Figure 5:**
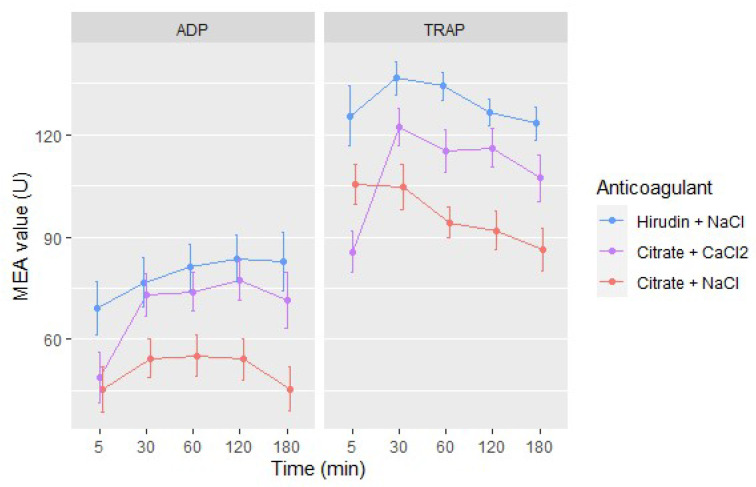
Mean MEA values (U) according to the time elapsed between blood draw and testing. Blue lines represent tests performed with blood samples anticoagulated with hirudin and diluted with NaCl, purple lines with citrate and diluted with CaCl_2_ and red ones with citrate and diluted with NaCl. Bars represent 95% CI of the mean.

The effect of time from blood draw until MEA analysis is represented in Table 3 and on Figure 5. There was globally a statistically significant reduction in platelet reactivity when tests were performed within 5 min after blood draw, by comparison with tests performed afterwards. However, those reductions were clinically acceptable for hirudin blood (the observed mean differences were lower than predetermined CD (i.e., 19% for ADP tests and 12% for TRAP tests)). We observed a trend toward a reduction in platelet reactivity as time from blood draw passed, which became statistically significant after 60, 120 or 180 min, depending on the test performed and the anticoagulant used (Table 3); those differences remained however clinically acceptable until 180 min for ADP tests (with all anticoagulants) and for TRAP tests with hirudin blood and until 120 min for TRAP tests performed with citrate blood.

MEA values were also significantly lower when citrate was used as anticoagulant (either diluted with CaCl_2_ 3 mM or NaCl 154 mM) by comparison with hirudin, for both MEA tests (ADP and TRAP; *p* < 0.001, LR tests) (Figure 5), except for ADP tests performed 30 min after blood draw if CaCl_2_ is used to dilute blood samples (Table 4). However, the differences observed between hirudin and citrate samples diluted with CaCl_2_ were clinically acceptable between 30 and 180 min after blood draw for ADP tests, and at 30 and 120 min for TRAP tests.

Finally, at baseline, there was a weak association between lower platelet counts (measured with EDTA blood) and reduced platelet reactivity for ADP test (R^2^ = 0.30, *p* < 0.001, Student *t*-test) and TRAP test (R^2^ = 0.07, *p* = 0.04, Student *t*-test).

### 3.4. Platelet Clumps

**Figure 6:**
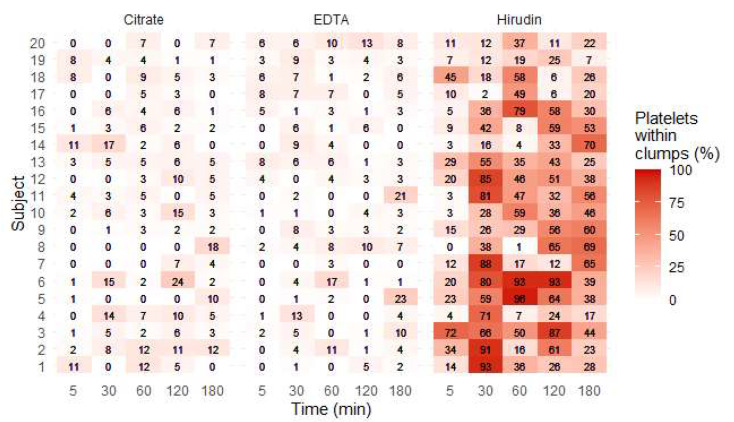
Percentage of platelets within clumps (%) for each subject, at each time point and for each anticoagulant.

In hirudin blood, there was an eight- and a tenfold increase in percentage of platelets within clumps (measured by optical microscopy (OM)), by comparison with citrate and EDTA blood, respectively (*p* < 0.001, LR test; Figure 6). This percentage increased mainly until 30 min (Figure S4). Of note, we only observed a weak correlation between the percentage of platelets within clumps measured by OM and Qflags for platelet clumps generated by the Sysmex analyzer (Spearman’s correlation coefficient 0.47).

## 4. Discussion

To the best of our knowledge, this is the first study assessing the effect of anticoagulants in the sample tubes at five time points (up to 180 min) after blood draw on platelet count, indices and function (impedance aggregometry in whole blood), and to evaluate the potential relationship of the presence of platelets clumps with the results of those tests as well. On the whole, we confirm the effect of preanalytical variables when performing platelet tests and therefore the importance of precisely specifying the preanalytical conditions used when determining decision thresholds in clinical trials.

There are two main preanalytical issues regarding platelet investigation in the laboratory, related to timing and anticoagulation: platelet changes (swelling, structural alterations) and clumping. In our study we refer to clumping, which can be agglutination or genuine aggregation, if it is mediated by fibrinogen binding to activated glycoprotein IIb-IIIa (GPIIb-IIIa). Regarding platelet enumeration, EDTA is the most appropriate anticoagulant in the vast majority of circumstances, but this is at the expense of structural changes. EDTA is inappropriate for platelet function testing, except for ristocetin-induced agglutination with platelet rich plasma [26]. When facing artefactual agglutination in EDTA blood, hirudin blood is not an option at all. Regarding platelet function testing, hirudin anticoagulation is by contrast an attractive option because calcium and magnesium levels are kept at their physiological levels, and hirudin is recommended for MEA. However, the occurrence of clumping raises the question of potential sequelae for functional investigations in such a milieu (i.e., hirudin whole blood).

Thus, we have chosen to start the discussion with the crucial issue of clumping, which we have attempted to quantitatively estimate with OM examination of blood smears.

### 4.1. Platelet Clumps

Platelet clumps were far more frequent when hirudin was used in sample tubes, by comparison with K_2_-EDTA and citrate. They formed mainly during the first 30 min after blood draw (Figure S4). The higher propensity of platelets to clump in hirudin whole blood has already been reported and was also found to persist over time, from 30 min to 24 h after blood draw [27]. Of note, the measured percentage of platelets within clumps was highly variable over time, probably partly reflecting the analytical variability (observer-dependent) associated with OM method. However, the formation of platelet clumps can be partly spontaneously reversible, mainly with low concentrations of activators and with normal calcium concentrations, such as in hirudin tubes [28]; this could explain the diminution of clumps percentage over time observed in some samples. This is consistent with the fact that for some subjects, the decrease in platelets within clumps percentage over time was accompanied with an increase in platelet count (Figure 3).

### 4.2. Platelet Count

The most stable platelet count over the 3-h study time-interval (room temperature) was obtained with EDTA. This is consistent with a previous study performed with EDTA blood, which failed to identify a significant reduction in platelet count over a period of 180 min after blood draw [29]. However, we identified a progressive relevant reduction in measured platelet count when citrate or hirudin was used as anticoagulant. This could be explained, partly at least, by platelet clumping. The effect was more pronounced for blood drawn into hirudin tubes when clumping was also more likely. Therefore, hirudin tubes should be avoided for the measurement of platelet count, unless the analysis is performed immediately after blood draw. Of note, platelet count was also higher when sample tubes were anticoagulated with EDTA, by comparison with hirudin and citrate, despite correction for dilution of the sample by liquid citrate. At baseline, this finding was only statistically significant for impedance platelet count and had already been reported [30]. Surprisingly, platelet count was lower when analyzed by the so-called optical method, compared to fluorescence method, although both methods are actually based on fluorescence counting (PLT-O are counted on the RET channel and PLT-F on a dedicated channel with different reagents). We also identified that impedance measurement method was more sensitive to the presence of platelet clumps than optical and fluorescence methods.

Overall, these results are in line with the guidelines of the International Council for Standardization in Haematology, which recommend the use of K_2_- or K_3_-EDTA tubes for the analysis of complete blood count and derived parameters [15]. Citrate tubes may be used for platelet counting in case of EDTA-induced pseudothrombocytopenia, but the clinician should be aware that the utilization of those tubes is associated with lower platelet counts, even if the dilution is corrected.

### 4.3. Platelet Indices

As previously described by other authors, we identified a significant increase in MPV over time since blood draw, occurring mainly during the first 30 min after blood draw. Citrate samples were associated with significant smaller MPVs than hirudin and EDTA samples, as previously outlined by Lancé et al. [31]. This could be explained by differences in blood resistivity induced by the different anticoagulants or by a lower degree of platelet activation during blood draw and processing in citrate tubes [32,33]. Indeed, Nishioka et al. identified a higher MPV increase in association with a higher degree of platelet activation after agitation of EDTA blood, by comparison with citrate blood [33]. All these changes were clinically significant (i.e., higher than the critical difference – CD, calculated at 5%). Of note, as previously reported, we found that, at baseline, higher platelet counts were associated with lower MPV, for all three anticoagulants [34,35]. Conservation of mass has been suggested to explain this finding, stating that a higher number of platelets can be produced if their volume is reduced [34].

The modification of MPV by the anticoagulant of the sample tube has been repeatedly reported and depends on the type and concentration of anticoagulant used [1,36]. This effect is due to platelets swelling caused by the anticoagulant. However, the exact mechanism is not fully understood and could include chelation of platelet membrane calcium ions by the anticoagulant [37]. This increase in platelet volume over time has been reported mainly during the first 5 min of exposure to anticoagulants [36]. Therefore, the anticoagulant used and the time elapsed before MPV measurement should be standardized within a same laboratory and reference ranges determined accordingly, especially when this index is needed for clinical decision.

Of note, swelling of platelets secondary to the anticoagulant has been reported to depend on the method of assessment of MPV: MPV increased over time when assessed by impedance method but decreased when assessed optically because of a reduced platelet density secondary to platelet swelling [36]. A previous study also identified an initial transient decrease of MPV for analyses performed immediately after blood draw, by comparison with analyses performed 30 min after; this finding was explained by an initial platelet sphering. MPV was then slightly increasing over the next three hours because of platelet swelling [29].

We also identified an increase in IPF measurement over time, except for EDTA samples. This could be partly a consequence of the increase in MPV over time previously outlined, as young platelets are larger than mature ones. However, IPF remained stable over time for EDTA samples despite an increased MPV. More likely, this could be a consequence of the formation of platelet clumps over time, erroneously measured as larger and denser immature platelets. This would be consistent with the more abundant formation of platelet clumps in hirudin tubes during the first 30 min after blood draw, accompanied by a more pronounced rise in IPF, compared to citrate and EDTA. These results are in agreement with a previous work performed on samples from patients with macrothrombocytopenia identifying an increase in IPF with platelet size and with the presence of platelet clumps [38]. Other authors proposed that this apparent increase in IPF as time since blood draw elapsed could be due to the release of RNA during degradation of young platelets during storage or to a true increase in young platelet count due to in vitro platelet division [39]. These results are of interest, as IPF measurement is often performed in the same blood only after the identification of thrombocytopenia [5]. The increase in IPF over that time-interval could lead to erroneously infer a peripheral mechanism for thrombocytopenia. As expected, at baseline, we also identified a significant association between low platelet count and increased IPF (*p* < 0.001), probably reflecting a compensatory increase in platelet production by the bone marrow when platelet count is low.

Our results somewhat differ from a previous study performed with blood drawn into K_2_-EDTA tubes stored at room temperature. The investigators identified an initial decrease in IPF during the first hour after blood draw and then a slow increase in IPF, becoming relevant after 12 h [40]. However, this decrease in IPF during the first hour was not present when samples were stored at 4 °C; IPF was actually increasing during this time-interval. Osei-Bimpong et al. also identified a progressive increase in IPF over time when blood samples were drawn in EDTA tubes and kept at 4 °C but without an initial drop [5,39].

Both MPV and IPF have been proposed as biomarkers of platelet turn-over and cardiovascular risk [1,41,42]. However, IPF seems less variable over time (especially when measured with EDTA samples). Therefore, this parameter should probably be preferred over MPV for that purpose.

### 4.4. Platelet Function

The anticoagulant used to keep blood non-clotted significantly influenced the result of both studied MEA tests (ADP, TRAP). The chelation of calcium ions by citrate resulted in reduced platelet reactivity. The adjunction of CaCl_2_ to the dilutent of citrate samples partially restored platelet reactivity, resulting in clinically acceptable differences for tests performed between 30 and 120 min after blood draw, by comparison with hirudin samples. This is in line with the results of Johnston et al., who also identified reduced platelet aggregability when citrate was used as anticoagulant instead of hirudin unless NaCl supplemented with 3 mM CaCl_2_ was used instead of mere NaCl to dilute citrate samples [14]. Therefore, if citrate tubes are used for MEA analysis, dilution with addition of CaCl_2_ 3 mM is preferable to reduce the difference of the measurement compared to hirudin tubes. However, MEA results still remained statistically lower than tests performed with hirudin blood. This could be explained by different plasma resistivity depending on the anticoagulant used [32]. Indeed, the reduction in platelet reactivity observed with citrate blood, by comparison with hirudin blood, has only been observed when platelet function was assessed with MEA [43]. On the opposite, other methods measured increased platelet function when citrate blood was used instead of hirudin blood (e.g., light transmission aggregometry, VerifyNow) or have not identified any statistically significant differences between either anticoagulant (e.g., PFA-100) [43].

We also identified a significant effect of the time elapsed between blood draw and MEA test. Platelet function was transiently reduced when assessed with ADP- and TRAP-MEA five minutes after blood draw. This could be a consequence of the activation of the blood sample during draw [44]. Then, platelet function remained quite stable until 120 min for ADP tests, before beginning to slightly decrease. For TRAP tests, platelet function peaked at 30 min followed by a slow decrease. This progressive reduction of platelet aggregability over time could be explained by incomplete platelet inhibition in the sample tubes, leading to progressive platelet activation and aggregation [45]. However, these changes remained clinically acceptable between 30 and 60 min after blood draw with citrate blood and all along the three hours study period with hirudin blood.

All in all, these results are consistent with previous studies. Indeed, most authors identified a reduced ADP-MEA during the first 30 min after blood draw, either with hirudin or citrate (dilution with NaCl) [44,46,47], even if this observation was not confirmed in other studies [14,48]. Then, platelet function was stable over at least three hours for citrate samples and until 60 to 240 min for hirudin samples [14,44,45,47,49]. A similar trend was observed for patients taking clopidogrel assessed by ADP-MEA, which however presented a more pronounced peak of platelet aggregability at 30 min, by comparison with clopidogrel-naive patients [50]. Few data are available for TRAP tests. Previous authors have not identified an initial decrease in platelet function assessed by TRAP-MEA performed within 30 min after blood draw, either with citrate (dilution with NaCl) or hirudin blood; platelet function remained stable over the 120 min study period [44]. In another study, platelet function was only decreasing from 120 min after blood draw in hirudin tubes [45]. Overall, the initial reduction of platelet reactivity observed for ADP and TRAP tests in our study is consistent with manufacturer’s recommendations and with several guidelines recommending waiting a minimum of 15 or 30 min after blood draw to perform platelet function testing [10,51].

Therefore, in order to reduce the variability of the measurement, it is reasonable to wait a minimum of 30 min after blood draw to perform MEA tests and then perform the analysis within two hours. Such specification of the timing to perform MEA, as well as anticoagulant used in sample tubes should be included in the guidelines proposing threshold values for MEA for clinical management.

### 4.5. Limitations

The study has several limitations. First, it was performed with healthy individuals. The impact of these preanalytical variables could be different for patients with platelet function defects or taking antiplatelet agents [52], for example. Additionally, in our cohort of healthy individuals, baseline platelet counts were higher than 100 G/L. This limits the extrapolation of our results to lower platelet counts. Secondly, this was a monocentric study, and platelet count and indices were determined with only one analyzer. Analyses performed with other devices could lead to different results. This is for example particularly important for MPV measurements because calibrators used to measure MPV, as well as methods themselves, are not standardized between analyzers [53]. Results could also be different when using platelet rich plasma instead of whole blood for platelet function testing [54]. Thirdly, we only evaluated three anticoagulants: in addition to EDTA and hirudin, recommended for platelet counting and MEA, respectively, we studied citrate, which is the most used anticoagulant for laboratory investigation of hemostasis. Heparin is also used in some centers for anticoagulation for platelet function analysis and could be considered as readily available in some settings (e.g., in the operating theater). However, it is generally not recommended for laboratory testing of primary hemostasis and has its own drawbacks (e.g., directly activates platelets and precludes blood smear examination by inducing staining artifacts); it has therefore not been evaluated in this study. Fourthly, the utilization of critical differences (CDs), derived from reference change values (RCVs), to judge whether differences observed between preanalytical conditions were clinically acceptable is debatable. Indeed, RCVs are used for the evaluation of the relevance of differences in serial measurement performed in the same individual and not for the evaluation of the influence of preanalytical variables. However, this index presents the advantage of being more objective than arbitrary thresholds and of taking into account the intrinsic variability of the measurement. Of note, the utilization of hydrodynamic focusing to line up cells in all channels of the Sysmex XN-20 could lead to the dissociation of the platelet clumps, at least some of them. This limits the reliability of the evaluation of the impact of platelet clumps measured by OM on parameters derived from Sysmex analyzers (i.e., platelet count, MPV and IPF). However, there was still a correlation between the proportions of platelets clumps evaluated by OM and with the Sysmex analyzer (Qflags), albeit moderate, even if the latter parameter is not intended to be used quantitatively. Finally, it would have been interesting to assess the impact of platelet clumps on MEA results. However, as the assessment of clumps was performed only with the sample tubes dedicated to platelet count and indices, and as platelet clumps percentages were highly variable, we decided not to perform this analysis.

## 5. Conclusions

This systematic, thorough study confirms the important effect of preanalytical variables on platelet count, platelet indices and platelet function measurement. The type of anticoagulant used in sample tubes and the time elapsed since blood draw had a statistically significant effect on most of the tests studied, even if the differences were not always clinically relevant. As currently recommended, EDTA should be preferred for platelet count and platelet indices measurement, since it provides more stable results over time. For platelet function analysis using MEA, citrate and hirudin can be used as anticoagulant in sample tubes, but values are not interchangeable. When using citrate tubes, dilution of the sample with NaCl 154 mM + CaCl_2_ 3 mM instead of mere NaCl 154 mM should be preferred to reduce the difference between both anticoagulants. MEA tests should be performed between 30 and 120 min after blood draw, and that time-interval should ideally be standardized within the same laboratory to reduce the variability associated with the measurement. Finally, precise preanalytical conditions should be specified when a decision threshold is proposed in clinical trials or guidelines. These thresholds should also be validated locally according to local settings (different analytical or preanalytical conditions such as different sample tubes) in order to prevent erroneous clinical decisions.

## Figures and Tables

**Table 1:** Effect of the time elapsed between blood draw and MPV measurement (expressed in fL) for each anticoagulant. Measurements at five minutes after blood draw are used as the reference. Mean difference, relative difference (%) and 95% confidence interval for the mean difference are given for each value.

	5 min	30 min (vs. 5 min)		60 min (vs. 5 min)		120 min (vs. 5 min)		180 min (vs. 5 min)	
EDTA	0	0.4 (3.8%) (0.3; 0.5)	***	0.6 (6.2%) (0.5; 0.7)	***	0.7 (7.2%) (0.6; 0.8)	***	0.8 (7.9%) (0.7; 0.9)	***
Hirudin	0	0.6 (6.2%) (0.5; 0.7)	***	0.7 (7.6%) (0.6; 0.8)	***	0.7 (8.0%) (0.6; 0.9)	***	0.9 (9.2%) (0.7; 1.0)	***
Citrate	0	0.6 (7.4%) (0.5; 0.8)	***	0.6 (6.9%) (0.5; 0.7)	***	0.8 (9.0%) (0.7; 0.9)	***	0.7 (8.4%) (0.6; 0.9)	***

*p* < 0.05 (*), < 0.01(**), < 0.001 (***). EDTA: ethylenediaminetetraacetic acid.

**Table 2:** Effect of the time elapsed between blood draw and IPF measurement (expressed as the percentage of young platelets to total number of platelets) for each anticoagulant. Measurements at five minutes after blood draw are used as the reference. Mean difference, relative difference (%) and 95% confidence interval for the mean difference are given for each value.

	5 min	30 min (vs. 5 min)		60 min (vs. 5 min)		120 min (vs. 5 min)		180 min (vs. 5 min)	
EDTA	0	0.1 (1.2%) (−0.5; 0.6)		0.1 (3.3%) (−0.4; 0.7)		0.1 (3.1%) (−0.4; 0.7)		0.2 (4.0%) (−0.4; 0.8)	
Hirudin	0	2.0 (51.2%) (1.4; 2.6)	***	2.3 (58.9%) (1.7; 2.9)	***	2.5 (54.8%) (1.9; 3.1)	***	2.7 (60.2%) (2.1; 3.1)	***
Citrate	0	0.5 (19.1%) (−0.05; 1.1)		0.7 (18.5%) (0.1; 1.3)	*	0.9 (23.4%) (0.3; 1.5)	**	0.9 (22.4%) (0.3; 1.5)	**

*p* < 0.05 (*), < 0.01(**), < 0.001 (***).

**Table 3:** Mean differences in MEA results at the five time points (30 min being the reference) for both activators and under the three anticoagulant conditions. MEA values are expressed in arbitrary units. Mean difference, relative difference (%) and 95% confidence interval for the mean difference are given for each value.

		5 min (vs. 30 min)		30 min	60 min (vs. 30 min)		120 min (vs. 30 min)		180 min (vs. 30 min)	
ADP	Citrate + NaCl	−9 (−19.9%) (−15; −3)	**	0	1 (3.0%) (−5; 7)		−0.2 (0.1%) (−6; 6)		−9 (−17.1%) (−15; −3)	**
	Citrate + CaCl_2_	−24 (−35.5%) (−30; −18)	***	0	1 (2.6%) (−5; 7)		4 (7.5%) (−2; 10)		−2 (1.0%) (−8; 4)	
	Hirudin	−7 (−10.0%) (−14; −1)	*	0	4 (7.0%) (−2; 10)		7 (11.6%) (1; 13)	*	6 (10.0%) (−0.01; 12)	
TRAP	Citrate + NaCl	1 (1.3%) (−6; 8)		0	−10 (−9.2%) (−17; −3)	**	−13 (−11.8%) (−20; −6)	***	−18 (−17.2%) (−25; −11)	***
	Citrate + CaCl_2_	−36 (−29.5%) (−44; −29)	***	0	−7 (−5.8%) (−14; 0.3)		−6 (−4.9%) (−13; 1)		−15 (−12.1%) (−22; −8)	***
	Hirudin	−11 (−8.1%) (−18; −4)	**	0	−2 (−1.2%) (−9; 5)		−10 (−6.8%) (−17; −3)	**	−14 (−9.2%) (−21; −6)	***

*p* < 0.05 (*), *p* < 0.01 (**), *p* < 0.001 (***).

**Table 4:** Mean differences in MEA results between the anticoagulants at the five time points. MEA values are expressed in arbitrary units with the two activators ADP and TRAP. Mean difference, relative difference (%) and 95% confidence interval for the mean difference are given for each value.

		5 min		30 min		60 min		120 min		180 min	
ADP	Citrate + CaCl_2_ (vs. Hirudin)	−20 (−30.3%) (−26; −14)	***	−4 (−3.0%) (−10; 2)		−7 (−6.3%) (−13; −1)	*	−6 (−6.7%) (−12; −0.4)	*	−11 (−8.3%) (−17; −5)	***
	Citrate + CaCl_2_ (vs. + NaCl)	3 (12.0%) (−3; 9)		19 (41.5%) (13; 25)	***	19 (41.9%) (13; 25)	***	23 (52.1%) (17; 29)	***	26 (76.7%) (20; 32)	***
	Citrate + NaCl (vs. Hirudin)	−24 (35.5%) (−30; −18)	***	−22 (−29.2%) (−28; −16)	***	−26 (−32.3%) (−32; −20)	***	−29 (−36.0%) (−36; −23)	***	−37 (−44.6%) (−43; −31)	***
TRAP	Citrate + CaCl_2_ (vs. Hirudin)	−40 (−30.1%) (−47; −33)	***	−14 (−9.9%) (−21; −7)	***	−19 (−14.0%) (−26; −12)	***	−11 (−8.0%) (−18; −3)	**	−15 (−12.3%) (−23; −8)	***
	Citrate + CaCl_2_ (vs. + NaCl)	−20 (17.5%) (−27; −13)	***	17 (18.5%) (10; 25)	***	21 (23.4%) (14; 28)	***	24 (27.9%) (17; 31)	***	21 (27.0%) (14; 28)	***
	Citrate + NaCl (vs. Hirudin)	−20 (−14.0%) (−27; −13)	***	−32 (−23.4%) (−39; −25)	***	−40 (−29.9%) (−47; −33)	***	−35 (−27.5%) (−42; −28)	***	−37 (−29.6%) (−44; −29)	***

*p* < 0.05 (*), *p* < 0.01 (**), *p* < 0.001 (***).

## Supplementary Materials

The following are available online at https://www.mdpi.com/2077-0383/9/8/2515/s1. Figure S1: Changes in platelet count over time between blood draw and analysis, Figure S2: Changes in mean platelet volume and of immature platelet fraction over time between blood draw and analysis for each anticoagulant, Figure S3: Changes in platelet function (assessed by MEA) over time elapsed between blood draw and analysis, Figure S4: Percentage of platelets within clumps over time for each anticoagulant, Table S1: Comparison of the mean differences in platelet counts at the five time points, Table S2: Comparison of the mean differences in platelet counts between the anticoagulants at the five time-points, Table S3: Effect of the method of measurement on platelet count for each anticoagulant, Table S4: Comparison of the effect of the anticoagulants on MPV measurement at the five time-points, Table S5: Comparison of the effect of the anticoagulants on IPF measurement at the five time-points.
