# Supplementary material for: Effects of Time-Interval since Blood Draw and of Anticoagulation on Platelet Testing (Count, Indices and Impedance Aggregometry): A Systematic Study with Blood from Healthy Volunteers
Source: J Clin Med. 2020 Aug 4;9(8):2515. doi: 10.3390/jcm9082515 (PMC7465339; doi:10.3390/jcm9082515)
Supplement: Supplementary file 1 [file jcm-09-02515-s001.pdf]

Table S1. Comparison of the mean differences in platelet counts (Sysmex XN - G/L, any method) at the five time points. Counts at five minutes after blood draw are used as the reference. Results are presented for each anticoagulant (corrected for the dilution when appropriate). Mean difference, relative difference (%) and 95% confidence interval for the mean difference are given for each value. *p* < 0.05 (*), < 0.01(**), < 0.001 (***).

	5min	30min (vs. 5min)		60min (vs. 30min)		120min (vs. 30min)		180min (vs. 30min)	
Citrate	0	-7 (-2.4%) [-18;5]		-19 (-6.8%) [-30;-7]	***	-28 (-10.3%) [-40;-17]	***	-35 (-13.2%) [-47;-24]	***
EDTA	0	2 (0.8%) [-9;14]		4 (1.4%) [-8;15]		4 (1.5%) [-7;16]		3 (1.2%) [-8;15]	
Hirudin	0	-58 (-22.6%) [-69;-46]	***	-79 (-29.9%) [-91;-68]	***	-98 (-37.0%) [-109;-86]	***	-113 (-43.3%) [-125;-101]	***

Table S2. Comparison of the mean differences in platelet counts (Sysmex XN - G/L) between the anticoagulants at the five time-points. Results are given for each method, either impedance, or so-called optical (RET channel), or fluorescence dedicated to platelet counting (corrected for the dilution when appropriate) and each time point. Mean difference, relative difference (%) and 95% confidence interval for the mean difference are given for each value. p < 0.05 (*), < 0.01(**), < 0.001 (***).

		5min		30min		60min		120min		180min	
**Impedance**	Citrate (vs. EDTA)	-31 (-11.0%) [-48;-13]	***	-37 (-12.6%) [-55;-20]	***	-49 (-16.5%) [-66;-31]	***	-59 (-19.8%) [-77 :-42]	***	-64 (-21.9%) [-82 ;-47]	***
	Hirudin (vs. EDTA)	-23 (-8.2%) [-41;-5.6]	*	-112 (-37.9%) [-129;-94]	***	-130 (-43.9%) [-147 ;-112]	***	-149 (-50.4%) [-116;-131]	***	-159 (-54.9%) [-177;-141]	***
	Hirudin (vs. Citrate)	8 (-3.2%) [-10;25]		-74 (-29.4%) [-92;-57]	***	-81 (-33.4%) [-99;-64]	***	-89 (-38.2%) [-107;-72]	***	95 (-41.6%) [-113;-77]	***
**Optical**	Citrate (vs. EDTA)	-9 (-3.4%) [-26;8]		-20 (-7.3%) [-37;-2]	*	-35 (-13.2%) [-52;-17]	***	-44 (-16.6%) [-62;-27]	***	-50 (-18.9%) [-68;-33]	***
	Hirudin (vs. EDTA)	-9 (-3.6%) [-27;8]		-54 (-20.4%) [-72;-37]	***	-81 (-30.8%) [-99;-64]	***	-98 (-36.7%) [-115;-80]	***	-111 (-42.5%) [-129;-94]	***
	Hirudin (vs. Citrate)	-0.2 (-0.1%) [-18;17]		-34 (-14.5%) [-52;-17]	***	-47 (-20.2%) [-64;-29]	***	-53 (-23.9%) [-71;-36]	***	-61 (-28.7%) [-78;-43]	***
**Fluorescence**	citrate (vs. EDTA)	-3 (-1.0%) [-21;14]		-14 (-4.8%) [-32;3]		-26 (-9.1%) [-44;-9]	**	-37 (-12.3%) [-54;-19]	***	-45 (-15.4%) [-62;-27]	***
	hirudin (vs. EDTA)	-12 (-4.2%) [-30;5]		-60 (-20.4%) [-77;-42]	***	-82 (-27.9%) [-99;-64]	***	-105 (-35.8%) [-122;-87]	***	-124 (-43.1%) [-142;-107]	***
	Hirudin (vs. Citrate)	-9 (-3.2%) [-26;9]		-46 (-16.6%) [-63;-28]	***	-55 (-20.9%) [-73 ;-38]	***	-68 (-26.7%) [-85;-50]	***	-80 (-32.4%) [-97;-62]	***

Table S3. Effect of the method of measurement on platelet count (G/L) for each anticoagulant (corrected for the dilution when appropriate). Results are presented as mean difference, relative difference (%) with 95% confidence interval for the mean difference. p < 0.05 (*), < 0.01(**), < 0.001 (***).

	Impedance vs. Fluorescence		Optical vs. Fluorescence		Impedance vs. Optical	
Citrate	-25.7 (9%) [-36 ;-15]	***	-34 (12.79%) [-44 ;-23]	***	8 (4.60%) [-2 ;18]	*
EDTA	-3 (0.60%) [-13;8]		-27 (9.41%) [-38 ;-17]	***	24 (9.80%) [14;35]	***
Hirudin	-40 (20.50%) [-51;-30]	***	-21 (-11.00%) [-32;-11]	***	-19 (11.13%) [-29;-8]	***

Table S4. Comparison of the effect of the anticoagulants on MPV measurement (expressed in fL) at the five time-points. Mean difference, relative difference (%) and 95% confidence interval for the mean difference are given for each value. p < 0.05 (*), < 0.01(**), < 0.001 (***)

	5min		30min		60min		120min		180min	
Citrate (vs. EDTA)	-0.8 (9.9%) [-0.9;-0.7]	***	-0.5 (4.7%) [-0.6;-0.4]	***	-0.8 (7.3%) [-0.9;-0.7]	***	-0.7 (6.4%) [-0.8;-0.6]	***	-0.8 (7.5%) [-0.9;-0.7]	***
Hirudin (vs. EDTA)	-0.01 (0.04%) [-0.1;0.1]		0.2 (2.3%) [-0.1;0.3]	***	0.1 (1.2%) [-0.01;0.2]		-0.04 (0.5%) [-0.1;0.1]		0.1 (0.9%) [-0.02;0.2]	
Citrate (vs. Hirudin)	-0.8 (7.8%) [-0.9;-0.6]	***	-0.7 (6.4%) [-0.8;-0.6]	***	-0.9 (8.3%) [-1.0;-0.8]	***	-0.7 (6.6%) [-0.8;0.6]	***	-0.9 (8.3%) [-1.0;-0.8]	***

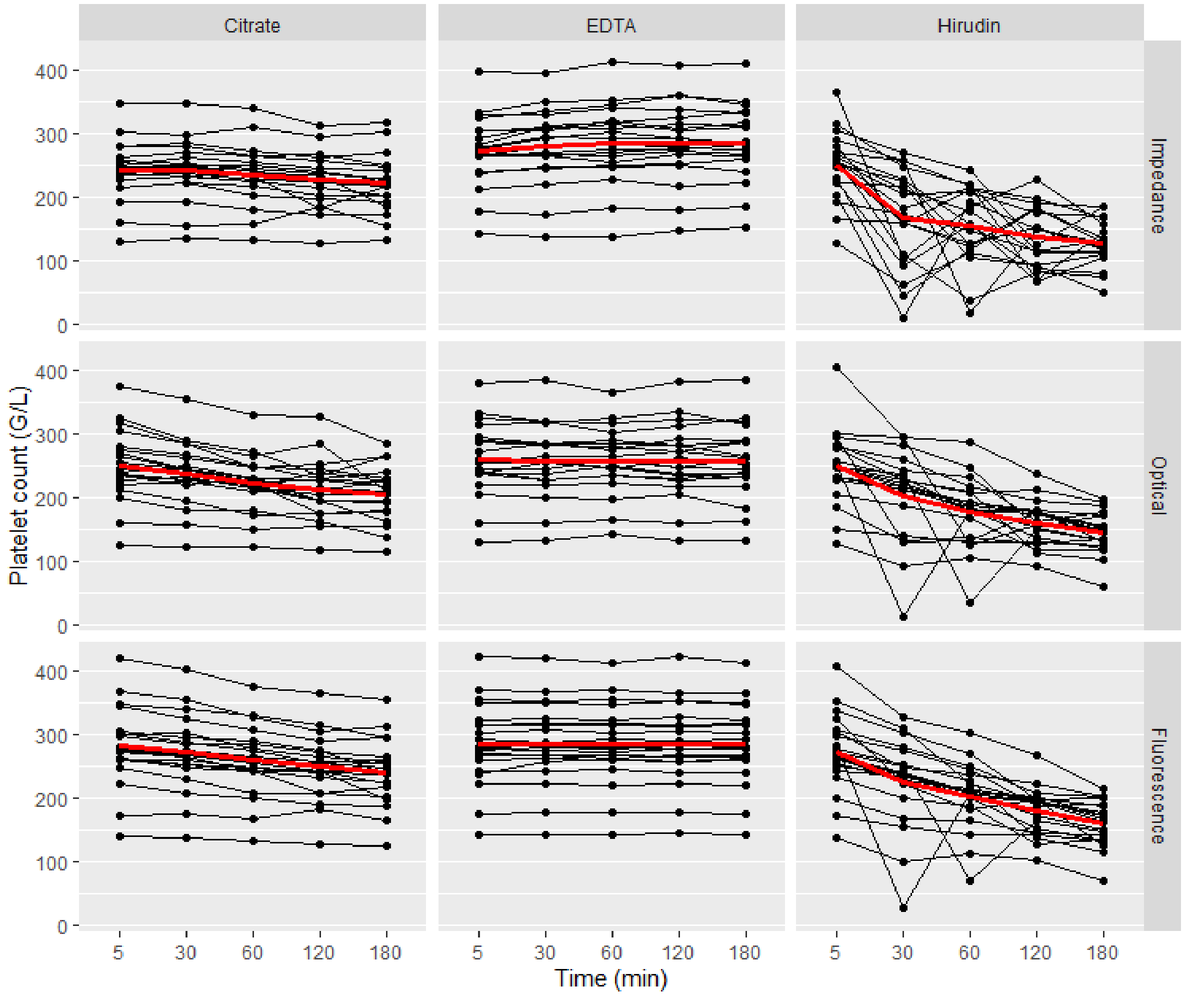

Figure S1. Changes of platelet count over time between blood draw and analysis. Each line represents a different patient. Mean platelet count over time is represented in red.

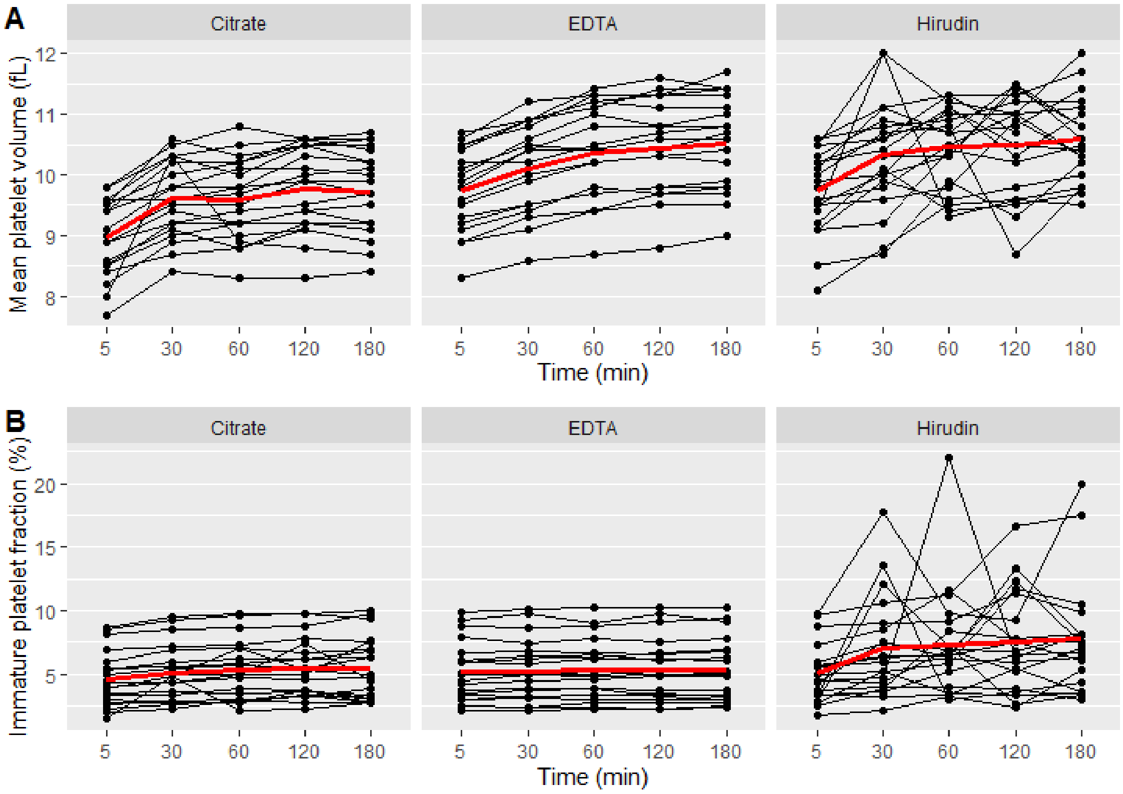

Figure S2. Changes of mean platelet volume (**A**) and of immature platelet fraction (**B**) over time between blood draw and analysis for each anticoagulant. Each line represents a different patient. Mean IPF over time is represented in red.

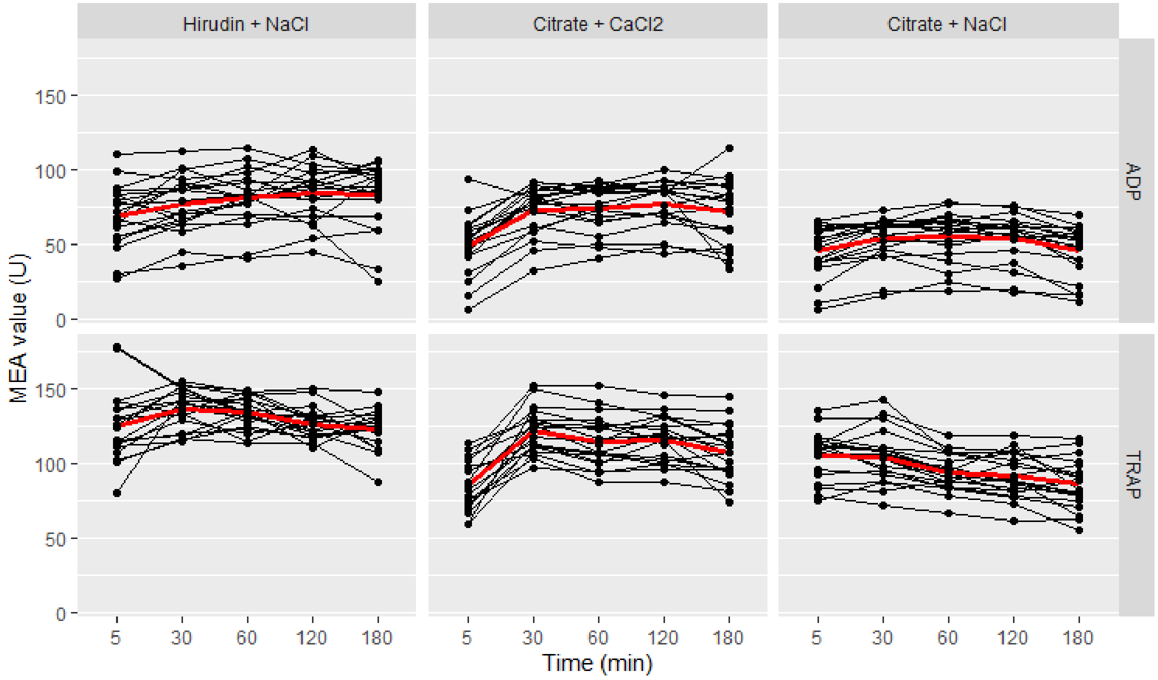

Figure S3. Changes in platelet function (assessed by MEA) over time elapsed between blood draw and analysis. Each line represents one subject. Mean MEA value over time is represented in red.
MEA: multiple electrode aggregometry.

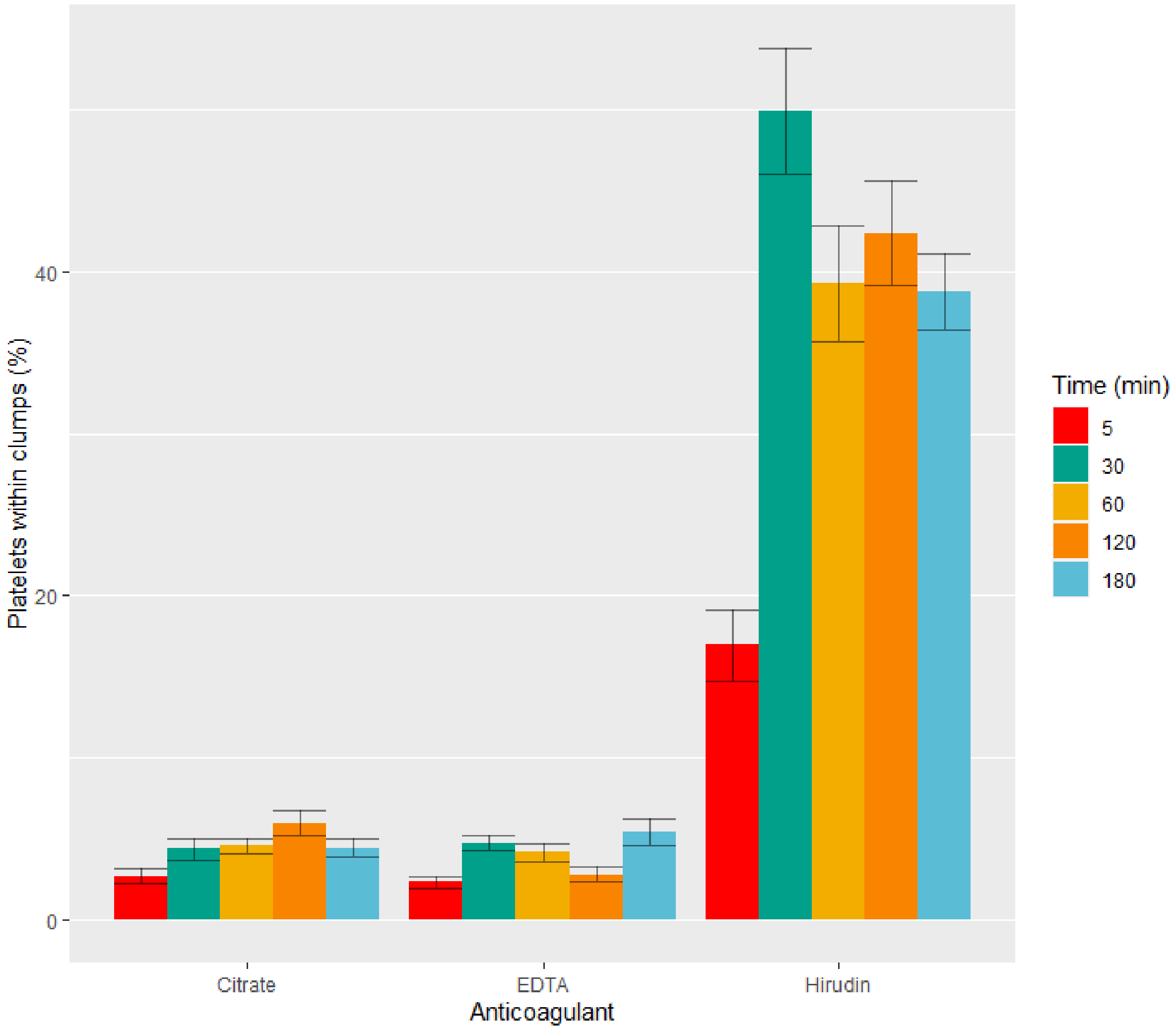

Figure S4. Mean percentage of platelets within clumps over time for each anticoagulant. Error bars represent standard deviations.
